# Adaptive Immune Responses Contribute to Post-ischemic Cardiac Remodeling

**DOI:** 10.3389/fcvm.2018.00198

**Published:** 2019-01-10

**Authors:** Icia Santos-Zas, Jérémie Lemarié, Alain Tedgui, Hafid Ait-Oufella

**Affiliations:** ^1^INSERM UMR-S 970, Sorbonne Paris Cité, Paris Cardiovascular Research Center – PARCC, Université Paris Descartes, Paris, France; ^2^UMR_S 1116, Université de Lorraine, Inserm, DCAC, Centre Hospitalier Régional Universitaire de Nancy – Réanimation Médicale – Hôpital Central, Nancy, France; ^3^AP-HP (Assistance Publique-Hôpitaux de Paris), Hôpital Saint-Antoine, Sorbonne Université, Paris, France

**Keywords:** T lymphocytes, B lymphocytes, dendritic cells, antibodies, cardiovascular disease, myocardial infarction

## Abstract

Myocardial infarction (MI) is a common condition responsible for mortality and morbidity related to ischemic heart failure. Accumulating experimental and translational evidence support a crucial role for innate immunity in heart failure and adverse heart remodeling following MI. More recently, the role of adaptive immunity in myocardial ischemia has been identified, mainly in rodents models of both transient and permanent heart ischemia. The present review summarizes the experimental evidence regarding the role of lymphocytes and dendritic cells in myocardial remodeling following coronary artery occlusion. Th1 and potentially Th17 CD4^+^ T cell responses promote adverse heart remodeling, whereas regulatory T cells appear to be protective, modulating macrophage activity, cardiomyocyte survival, and fibroblast phenotype. The role of CD8^+^ T cells in this setting remains unknown. B cells contribute to adverse cardiac remodeling through the modulation of monocyte trafficking, and potentially the production of tissue-specific antibodies. Yet, further substantial efforts are still required to confirm experimental data in human MI before developing new therapeutic strategies targeting the adaptive immune system in ischemic cardiac diseases.

## Epidemiology

Chronic diseases have emerged as main contributors to global mortality and morbidity (1). By 2015, the number of deaths related to cardiovascular disease (CVD) (ischemic heart disease, stroke, and valvular heart disease) has reached 17.5 million, more than 7 million being attributed to coronary artery disease and 6 million to stroke. In the future, CVD will be the largest contributor to global mortality, ahead of infectious diseases and maternal and perinatal conditions (2, 3).

**Figure 1:**
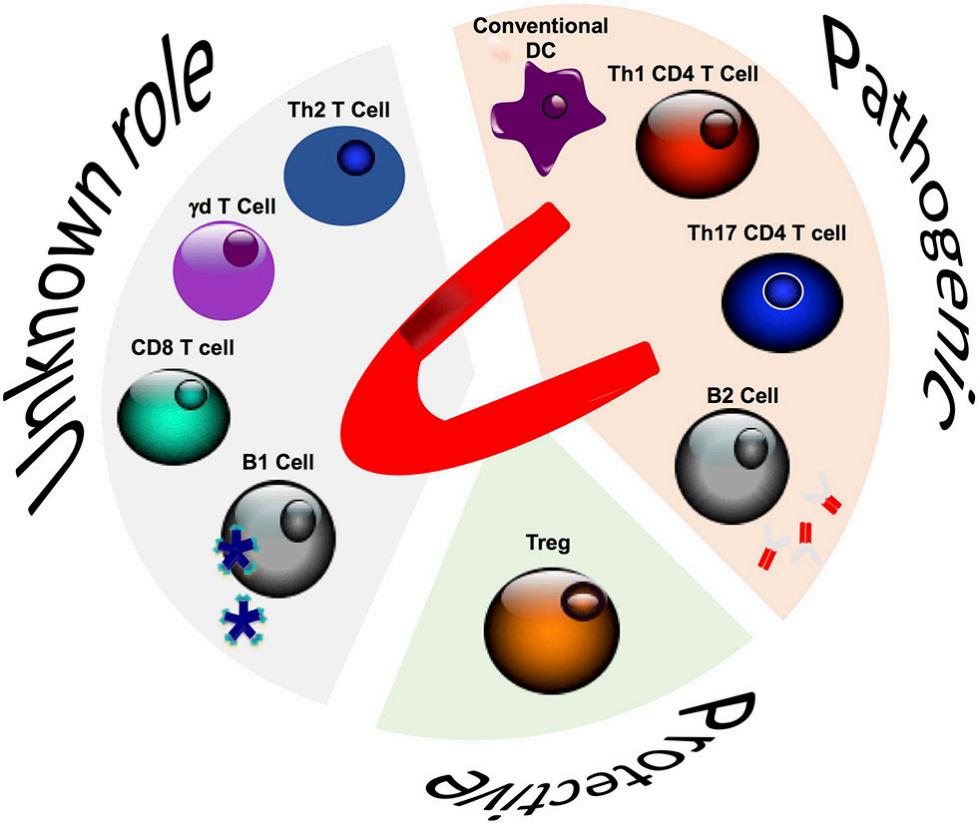
Role of adaptive immune cells in post-ischemic cardiac remodeling.

In the context of myocardial infarction (MI), a progressive decrease in early mortality over time has been described in the United States (4) and Europe (5). This change is explained by improvements in the management of acute MI patients, including the more frequent coronary reperfusion using fibrinolysis or primary percutaneous coronary intervention (PCI). As the number of survivors following MI patients increases, the prevalence of CVD with its associated complications is also raising (6). Acute myocardial ischemia and reperfusion following primary PCI are responsible for cardiac tissue damages that lead to deleterious myocardial remodeling and heart failure. Data from the Framingham Heart Study show that the incidence of heart failure at Day 30 after MI rose by two-fold from 1970–1979 period to 1990–1999 period whereas mortality at Day 30 after MI declined (7). Therefore, in association with anti-thrombotic medication and re-opening of the culprit coronary artery, there is urgent need to improve our understanding of the pathophysiological mechanisms that promote adverse ischemic cardiac remodeling and heart failure. There is accumulating evidence in rodent models that immune-inflammatory responses are involved in deleterious cardiac post-ischemic remodeling. This review aims to summarize clinical and experimental evidence regarding the role of immune-inflammatory responses in myocardial remodeling following MI (Figure 1).

## Innate Immunity and Myocardial Infarction

MI injury due to atherosclerotic plaque disruption and thrombosis is the first cause of heart failure (8). Innate immune responses contribute to the complications of atherosclerosis and consecutive acute MI. Monocytes/macrophages and neutrophils are implicated in adverse myocardial remodeling following MI and might promote heart failure. In human and rodents models of MI, interruption of coronary blood flow leads to rapid cardiomyocytes death in the ischemic myocardium. Thereafter, inflammatory signals allow recruitment of inflammatory cells, which profoundly alter left ventricle (LV) structure and function through their impact on extracellular matrix degradation/deposition, clearance of dead cardiomyocytes and their debris, and the resolution of inflammation. In mice, large amount of neutrophils infiltrate the heart tissue within the first day, followed by a biphasic infiltration of monocyte subsets Ly6-C^high^ and Ly6C^low^. Ly6-C^high^ monocytes dominate the acute phase of injury during the first 4 days and contribute to adverse tissue remodeling, while Ly6C^low^ monocytes become prevalent thereafter and, along with resident cardiac macrophages (9), play a protective role in tissue healing and neovascularization (10). Similar mechanisms may also operate in humans (11).

## CD4+ T Cells

Conversely to innate immune responses, the contribution of lymphocytes to post-MI inflammatory response and repair has been the subject of very few studies for the last 2 decades. Among the adaptive immune actors, CD4^+^ helper T lymphocytes, which interact with antigens presented on MHC-II molecules (expressed on antigen-presenting cells) have been the most regularly investigated (12). CD4^+^ T cells are usually allocated into distinct subsets according to their phenotype polarization and distinct cytokine repertoire. Th1 and Th2 populations were first described and then, many additional Th subsets, named according to their cytokine production (Th17 cells, Th9, and Th22, respectively producing IL-17, IL-9, and Il-22) or function (regulatory Tregs), were uncovered (13).

### CD4+ T Cell Infiltration in Ischemic Heart Tissue

In the steady state (no myocardial injury), in mouse, flow cytometry-based absolute number of CD4^+^ T cells ranges between 10^3^ and 10^4^ per heart, depending on the digestion and leukocyte enrichment protocols used (14, 15). After permanent coronary occlusion, mimicking MI, this level increases by 10-fold, peaking at day 7 (15). In experimental models of myocardial ischemia/reperfusion, CD4^+^ T cell infiltration is lower (two-fold increase compared to sham operated animals) and peaks earlier at day 3. Based on histological observations, some authors reported CD4^+^ T cell recruitment in the heart even much earlier, during the first minutes after reperfusion (16). In human autopsies, infiltrating CD3^+^ T lymphocytes have been found in ischemic heart (17). In MI patients during the reperfusion procedure, a coronary artery gradient in CD4^+^ T cells count was found between the arterial and the venous blood, suggesting emigration of these cells from the arterial blood flow to the ischemic myocardium (18). Lymphocyte count drop was mainly observed among CCR7^+^ CD4^+^ T cells, suggesting that this chemokine receptor and its ligands CCL19 and CCL21 (expressed by endothelial cells) are involved in their recruitment within the myocardial vasculature (19).

### General Role of CD4+ T Cells in Post-ischemic Cardiac Remodeling

Experimental murine models provide convincing evidence that CD4^+^ T cell responses participate in post-MI heart remodeling, but their roles differ depending on the injury model considered: ischemia/reperfusion (IR) or permanent occlusion. In reperfused models, CD4^+^ T cells have been shown to promote IR-related cardiac damage. Using anti-CD4 depleting antibody in wild-type (WT) mice, as well as lymphocyte-deficient Rag1^−/−^ mice reconstituted with purified CD4^+^ T cells, Yang et al. first demonstrated the deleterious role of CD4^+^ T cells in modulating infarct size (16). Their recruitment, mainly from the spleen reservoir, can be prevented by blocking A2A or A2B adenosine receptors. Conversely, Hofmann et al. showed in a permanent artery coronary ligation model in mice that CD4^+^ T cells became activated in mediastinal lymph nodes within few days after MI and were required for collagen deposition, a protective mechanism against left cavity dilation and rupture (20, 21).

### Role of Th1/Th2 T Cells in Post-ischemic Cardiac Remodeling

For a long time, several seminal studies suggested that CD4+ naïve T cells polarize toward either Th1 or Th2 populations according to specific and mutually exclusive differentiation programs. IFN-γ and IL-12p70 triggered Th1 commitment, fully differentiated Th1 T cells being characterized by T-bet expression and IFN-γ production. IL-12p70 drives signal transducer and activator of transcription (STAT-4) and T-bet. In turn, T-bet promotes IFN-γ production and IL-12 receptor expression, while down regulating both IL-4 and IL-5 expression. Th1 CD4^+^ T cells are involved in immunity against pathogens, but have also been implicated in auto-immune and inflammatory diseases such as atherosclerosis (22). Dendritic cells (DCs) play a major role in Th2 differentiation through cytokine secretion (IL-6 and IL-13) and membrane-bound costimulation (OX40-OX40L) (23). Th2 cells regulate B cell-mediated humoral responses, especially against extracellular pathogens and also secrete several cytokines including IL-10, IL-4, IL-5, and IL-13. IL-4 induces in a STAT-6-dependent mechanism the expression of GATA-3, the Th2 differentiation transcription factor (24), which upregulates IL-4 and IL-5, and inhibits the production of IFN-γ. As a consequence, Th2 cells might counteract the Th1 responses.

After MI, the Th1/Th2 balance in the heart is skewed toward a Th1 phenotype, as shown by a 2-fold increase in IFN-γ producing CD4^+^ T cells, contrasting with an almost complete lack of CD4^+^ T cells that produce IL-4 (15). The prevailing contribution of Th1 subset was also established in resupplementation experiments. Reconstitution of immunodeficient Rag1^−/−^ mice with Ifn-γ^−/−^ CD4^+^ T cells did not recapitulate the detrimental effects of WT CD4^+^ T cells transfer in a transient myocardial ischemia model. Recently, Dectin-2, a receptor expressed on myeloid cells, has been shown to promote Th1 immune response through the increase of IL-12p70 production within the infarcted heart. Th1 polarization is associated with increased cardiomyocyte apoptosis, imbalanced extracellular matrix turnover and decreased myofibroblast differentiation leading to cardiac rupture (25). However, the role of IFN-γ is more complex, as it may also promote regulatory T cell activation and expansion (26).

In human, T cell profiling in the context of MI has been poorly investigated. Some studies have investigated a specific Th1 subpopulation, called CD4^+^CD28^null^, in cardiovascular diseases such as atherosclerosis and acute coronary syndromes. CD4^+^CD28^null^ T cells, which do not exist in mice, expand in several chronic inflammation diseases, but are almost undetectable in healthy individuals (27). CD4^+^CD28^null^ T cells secrete IFN-γ and TNF-α, as well as cytotoxic mediators (perforin and granzyme B) and are present in the blood in the context of acute coronary events during several months (28).

### Role of Th17 T Cells in Post-ischemic Cardiac Remodeling

IL-17A producing Th17 effector cells can be primed in heart-draining lymph nodes after MI by conventional type 2 DCs (29). However, their role in the context of MI has not been fully addressed yet. IL-17A has been shown to increase myocardial fibrosis in a rodent model of heart failure induced by infusion of isoproterenol (30). It is also noteworthy that more than 90% of IL-17A producing cells within the infarcted myocardium are γδ CD4- T cells (31). IL-17A is involved in late-stage ventricular remodeling after MI, by promoting sustained infiltration of neutrophils and macrophages, pro-inflammatory cytokine production, cardiomyocyte death, and fibrosis.

### Regulatory T Cells

**Figure 2:**
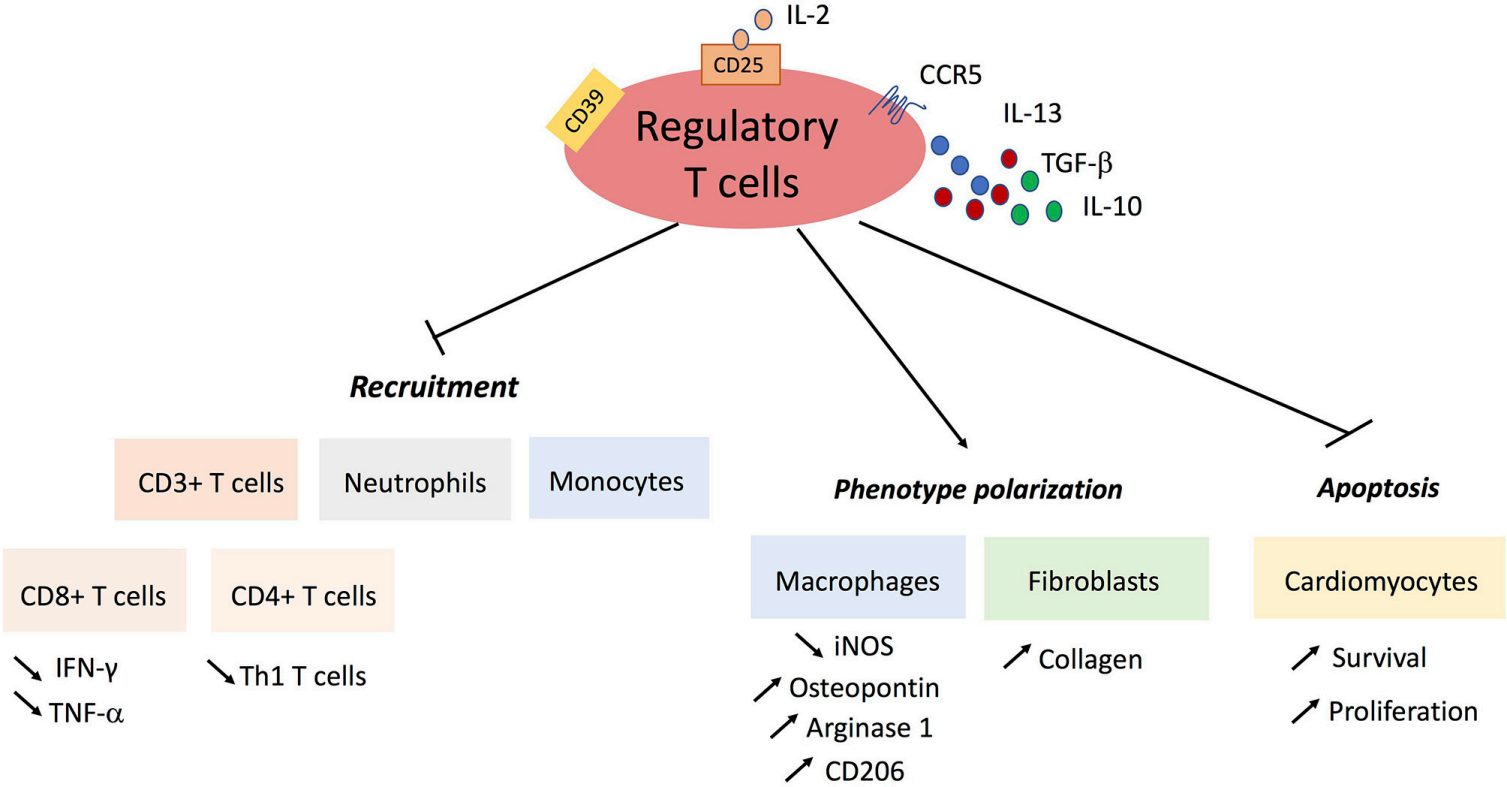
Protective mechanisms of regulatory T cells in post-ischemic cardiac remodeling.

CD4^+^ T subsets with immunosuppressive functions have also been described, the most important one named natural regulatory T cells (Tregs). Treg cells are generated in the thymus during fetal development and the first years of life, while induced Treg (iTreg) cells can developed later in the periphery from naïve CD4^+^ T cells. Treg cells express a specific transcription factor called FoxP3, for the forkhead/winged helix transcription factor, crucial for their development, and functions. Treg cells limit autoimmunity and maintain self-tolerance through the suppressing of activated effector T-cells, directly or through inhibition of antigen-presenting cells (32). Few “resident” Treg cells are present in the healthy myocardium, but they rapidly infiltrate the myocardium in the context of acute ischemia in a CCR5-dependent manner (33), peaking after 24 h in a reperfused model of MI (34) or day 7 in a permanent artery ligation model (35). Foxp3 mRNA local expression gradually increases during the first two weeks after MI (15). Almost all the studies have reported a beneficial role for Tregs in experimental MI models. Treg depletion using anti-CD25 depleting antibody-mediated or diphteria toxin-induced model worsens cardiac inflammation, infarct size and left ventricular dysfunction following MI. By contrast, Treg expansion (using anti-CD28 antibody administration) improves both survival and myocardial wound healing. Tregs limit pathogenic CD8^+^ and CD4^+^ T cells recruitment in the heart and shape the monocyte/macrophage polarization toward a pro-fibrotic phenotype through upregulation of Osteopontin, Arginase-1 and CD206. In addition, Treg expansion increased both procollagen α-1 (I) and procollagen α-1 (III) mRNA expression, as well as collagen I and collagen III protein levels in the heart tissue (35). The protective effect of Treg cells was at least partially related to membrane-bound ectonucleoidase (CD39) expression (36). At later stages, Tregs also interact with fibroblasts and promote a matrix-preserving cardiac fibroblast phenotype (34). Recently, it has been reported that Tregs can limit cardiomyocyte apoptosis, and can even induce their proliferation through the release of several soluble factors including IL-10 and IL-33 (37, 38) (Figure 2). In MI patients, no direct evidence is available regarding the contribution of Treg cells to myocardial healing. One study found a decrease in circulating Tregs after MI (39).

Several therapeutic strategies for Tregs expansion are currently under development. Anti-CD28 superagonistic antibodies that activate T-cells without TCR ligation (40), induced polyclonal Treg expansion *in vivo* and IL-10 overproduction (41). However, despite promising experiments results, the phase I trial of superagonistic anti-CD28 antibody in humans was stopped due to unexpected toxicity (42). Other strategies to promote Tregs, e.g., through anti-CD3 monoclonal antibody injection (43) or supplementation with low-dose IL-2 (44), may still be of interest. Villalta et al. have reported that administration of recombinant IL-2/anti-IL-2 mAb complex in dystrophic mice induced expansion of Treg *in vivo*, increased IL-10 tissue level and prevented muscle damage (45). Such an IL-2-based strategy is currently under investigation in MI patients (46).

### Antigen-Specific CD4+ T Cell Responses

Mechanisms driving CD4^+^ T helper polarization are mostly mediated by cytokines produced by antigen-presenting cells and other surrounding inflammatory actors. However, in the context of MI, whether lymphocytes are activated through TCR-mediated antigen recognition or unspecific inflammatory signals, including alarmin recognition by pattern recognition receptors (PRRs), remains controversial. In 1998, Maisel et al. reported that transferring splenocytes from MI rats in naïve healthy rats induced an autoimmune myocarditis with no evidence for immune cell infiltration in other organs, suggesting the development of self-reactive T cell clones against myocardial antigen after MI (47). Involvement of TCR engagement for CD4^+^ T cell activation was further demonstrated by Hofmann et al. using a model of permanent ligation in *Cd4*^−/−^*, MhcII*^−/−^, and *OT-II* mice. These latter mice have CD4^+^ T cells bearing a transgenic TCR for an irrelevant ovalbumin-derived peptide. The authors found that the 3 genotypes shared the same detrimental phenotype with impaired scar formation and decreased survival (21). Using several elegant genetically-modified mouse models, Van der Borght et al. reported an expansion of Tregs, Th1, and Th17 CD4^+^ T subsets in mediastinal lymph nodes through a TCR-mediated mechanism involving α-myosin heavy chain presented by conventional type 2 DCs (29). This specific self-antigen response has also been reported in models of experimental auto-immune myocarditis (48) and in chronic Chagas cardiomyopathy (49). Such autoreactive CD4^+^ T cell responses against heart derived- self antigens, including α-Myosin Heavy Chain (α-MyHC), could be explained by a defective tolerance induction against these antigens during fetal thymic selection. Lv et al. have reported that α-MyHC expression was not detectable in human thymus, a critical step to induce tolerance, and consequently α-MyHC–specific T-cells were found in the blood from healthy subjects (48).

## CD8+ T Cells

CD8^+^ T cells play a major role in immunity, directly killing virally infected or damaged cells. Activation of CD8^+^ T cells requires interaction of the TCR receptor with peptide presented by MHC class I molecules on antigen-presenting cells. CD8^+^ T cells are able to lyse target cells through the secretion of perforin and granzymes, and to a less extend through the engagement of membrane bound death-inducing ligands such as Fas-ligand.

The pathogenic role of CD8^+^ T cells has been identified in several experimental models of viral myocarditis. Heart tissue lesions are attenuated in *Cd8*^−/−^ mice infected with Coxsackie virus compared to wild-type animals, and reconstitution of these immunodeficient mice with purified CD8^+^ T cells worsens systolic LV dysfunction (50). The mechanisms that drive CD8^+^ T cells activation and expansion are complex, including CD4^+^ T cell-dependent mechanism through the release of IFN-γ and the production of IL-15 by different immune cell subsets (51). In addition, IL-21 receptor engagement on CD8^+^ T cells is also involved in myocardial damage in a murine model of viral myocarditis (50).

Little is known about the role of CD8^+^ T cells in acute ischemia. In a mouse model of stroke, CD8^+^ T cells have been shown to be recruited into the brain (52). In the same line, after ligation of the femoral artery in rodents, CD8^+^ T lymphocytes infiltrate the limb, induce CD4^+^ T cells recruitment in an IL-16-dependent manner and promote angiogenesis (53). In a model of MI in rats induced by permanent ligation of descending left coronary artery, a subset of CD8^+^ T cells expressing the angiotensin type 2 receptor (AT2R) infiltrates the peri-infarct zone and downregulates pro-inflammatory cytokines expression. These CD8^+^AT2R^+^ T cells have no cytotoxic activity, suggesting a potential cardioprotective role of this subset in the context of ischemia (53). Conversely, *in vitro*, CD8^+^ T cells isolated from MI rats have cytotoxic activity and directly kill healthy cardiomyocytes. CD8^+^ lymphocytes isolated from the same rat strain (Sprague–Dawley) induced significantly more myocyte death than lymphocytes isolated from another strain (Wistar), suggesting MHC class I- and antigen-specific cytotoxic response (54).

Human data regarding the role of CD8^+^ T cells in MI are scarce. In a small cohort of patients admitted for acute coronary syndrome, an increase in activated CD69^+^CD8^+^ T cells have been described, as well as increased soluble Fas Ligand and granzyme B levels in the blood at day 7 and 14 day after myocardial ischemia. A correlation between Granzyme B plasma levels and left ventricular end-diastolic diameter was reported, suggesting a role for this serine protease in deleterious LV remodeling after MI (55). Finally, an observational study has described an acute reduction in blood CD8^+^ T cell count within 1 h after coronary artery reperfusion, probably due to cell recruitment into the heart ischemic tissue. The drop of CD8^+^ T cells is more important in patients that develop heart microvascular obstruction (18). Overall, further studies are required to elucidate the role of CD8^+^ T populations in myocardial post-ischemic remodeling.

## B Cells

Conventional B-2 cells, the dominant B cell subset in spleen and lymph nodes, originate from the bone marrow and contribute to humoral and adaptive immune responses in a T-cell dependent manner. B-2 cell responses are highly specific but delayed (56). Conversely, B-1 cells is a minor B cell population present mainly in the peritoneal/pleural cavities and the spleen (5%), deriving from splanchno-pleural tissues, and fetal liver (57). B-1 cells are long-lived cells with reduced antigen affinity in comparison with B-2 cells. B-1 responses are rapid but poorly specific, secreting natural IgM antibodies in a T-cell independent manner. The IgM low affinity antibodies produced by B-1a cells can react with bacterial pathogens but also with oxidized lipid moieties (58).

More recently, specific B cell subpopulations with regulatory properties (Breg) were characterized (59). Breg populations share some functional (IL-10 production) and phenotypical [CD5+CD1d^high^ (60), Tim-1^+^ (61) characteristics with B-1 cells or Marginal Zone B cells (62)]. A new B-1 cell subset, innate response activator (IRA) B cells, has been identified (63), migrating from the peritoneal cavity to the spleen and producing granulocyte-macrophage colony-stimulating factor.

### B Cell Recruitment

B lymphocytes play critical roles in both innate and adaptive immune responses through antibody-dependent or independent mechanisms. Novel protective role for B cells in the immune response against bacterial pathogens has been found (63, 64). However, the contribution of B lymphocytes to the inflammatory response secondary to sterile injury, particularly post-ischemic injury is still poorly defined. In experimental MI in mice, B220^+^IgM^+^ B cells peaked in the heart tissue at day 5 after the onset of ischemia (65). CD20^+^ B cells have also been found in human heart biopsy from MI patients at day 1 and day 6, following coronary artery occlusion. The mechanisms that drive B cell recruitment and activation are yet under investigation but it appears that several myocardial auto-antigens (described below) recognized by B-cell receptor or, alternatively, pattern recognition receptors such as TLR-9 are involved (65).

### Humoral B Cell Responses

Plasma heart-specific antibodies have been detected in both rodents and patients with ischemic heart failure (66, 67). These autoantibodies target proteins from sarcomere (myosin, actin, and troponin) (67), cardiac receptors (anti-**β**1-adrenergic receptors) (68), and damage-associated epitopes (69). A positive relationship has been reported between anti-myosin antibody titers and infarct size on one hand, and between (70) anti-myosin antibody titers and prognosis in MI patients (67) on the other. Leuschner et al. found that cardiac remodeling was different in MI patients according to baseline anti-troponin auto-antibodies levels. A significant LV dilation was observed 6–9 months after MI in patients with, but not in patients without, detectable troponin auto-antibodies in the plasma (71).

Autoantibodies (named natural autoantibodies) could also be detected in healthy subjects before the onset of cardiac injury. Natural auto-antibodies can recognize damage-associated molecular patterns, including non-muscle myosin heavy chain II, and readily accumulate in wounded tissues, including ischemic heart (69, 72, 73). In experimental models of ischemia-reperfusion injury, natural IgMs have been shown to activate the complement cascade promoting inflammation in ischemic tissues (69, 73). B cell responses appear to be redundant in post-ischemic stroke injury, since B cell-deficient (μMT) mice showed no difference either in infarct size nor in clinical neurological disorder, as compared with wild type mice (74). The use of B cell-deficient μMT mice in the context of kidney ischemia/reperfusion has led to divergent results, with studies showing either protection (74, 75) or aggravation (76) in mice lacking B cells. The use of μMT mice should be interpreted with caution given the associated immune abnormalities in this model, and the fact that all B cell subsets, with potentially opposite functions, are absent in this strain. More recently, Keppner et al. used another mouse model to selectively study the role of (auto-) antibodies during post-ischemic heart context (77). The authors induced MI in agammaglobulinemic AID^−/−^μS^−/−^ mice that can produce functional B-cells, but cannot synthesize secretory IgM (μS^−/−^) or perform immunoglobulin class-switching (AID^−/−^). When compared to immunocompetent animals, agammaglobulinemic mice are characterized by a significant reduction in infarct size, in left ventricle dilation and improved cardiac function at day 56 post-MI, suggesting that antibodies are directly involved in ischemic heart failure (77). Heart tissue analysis showed less *Mmp9 mRNA* expression in the B cell-deficient group. Further studies are required before providing definitive conclusion regarding the role of immunoglobulins in post-ischemic cardiac remodeling.

### Cellular B Cell Responses

B cells have classically been thought to contribute to the immune response through antibody production after plasma cells differentiation. However, several studies in human and in rodents have found that genetic or pharmacologic B-cell depletion, in type 1 diabetes or rheumatoid arthritis, can regulate T-cell-mediated auto-immune diseases independently of antibody production, which suggests that the cellular functions of B cells are important in the regulation of the adaptive immunity (77). B cells secrete several cytokines, including B cell-derived lymphotoxin-α and TNF-α that control the development of follicular DCs and the formation of B cell follicles in the spleen. In MI, our group has identified a critical cellular role for mature B cells in left ventricular remodeling and function. At the acute phase of MI, the specific Ccl-7 production by mature B cells orchestrates monocyte mobilization from the bone marrow to the blood and *in fine* their recruitment into the ischemic heart (65). Specific deletion of CCL-7 production by B cells limits monocyte/macrophage infiltration in the ischemic heart, collagen deposition and reduces deleterious LV remodeling. In a French cohort of patients with MI (FAST-MI), plasma levels of CCL-7 were predictive of major adverse cardiovascular events, corroborating the experimental data. These recent results open promising new therapeutic area of ischemic heart failure using anti-CD20 depleting antibody in MI patients.

## Dendritic Cells

### Ontogeny and Subsets

DCs are potent key immunoregulators that orchestrate various types of inflammatory cells (78). DCs originate from CD34^+^ precursors of the myeloid lineage in the bone marrow (79) and after a circulating phase, they populate tissues close to epithelial area, where they act as sentinels of infection or injury. Different DC sublineages have been characterized in mice and humans (80, 81). Three major precursors of blood DCs have been described: Fms-like tyrosine kinase 3 (Flt3)^+^ pre-classical DCs (cDCs), colony-stimulating factor 1 receptor (CSF1R)^+^ monocytes, and Flt3^+^ plasmacytoid DCs (pDCs) (82). Monocytes can also become CD11b^+^ DCs, expressing DC associated antigens and the capacity to activate T cells (83).

After microbial infection or sterile injury, circulating monocytes and DC precursors from the bone marrow and the spleen reservoir differentiate into mature DCs and can modulate the immune system at the inflammatory site, such as priming of antigen-specific immune responses, induction of tolerance, and chronic inflammation (84). In the context of MI, DCs internalize locally released cardiomyocyte-derived antigens and migrate into mediastinal lymph nodes, where they present the antigen-derived peptides on both MHC class I and MHC class II molecules, after lysosomal degradation (85). CD4^+^ T cell priming requires TCR ligation by the cognate peptide–MHC complex, and membrane-bound costimulatory molecules, including CD28 and CD40 (86). Meanwhile, T cells are activated by locally released cytokines toward a Th1, Th2, or Th17 profile, and migrate to the inflamed tissue. Several subsets of DCs, expressing CD11c (87), Cd11b or CD103 (88), infiltrate the infarcted heart in experimental MI (87), and express markers of activation, including CD40 (88). GM-CSF, locally produced by endothelial cells, or resident and infiltrating leucocyte subsets, is involved in DC recruitment (89). The number of mature DCs in the infarcted heart correlates with LV dysfunction in rats with MI (89). However, the causative effect of infiltrating DCs subsets on LV remodeling and their origin in the post-infarction healing process remain unclear. Using transgenic mice expressing diphtheria toxin receptor on CD11c^+^ DCs, it has been shown that DC depletion enhances inflammatory cytokine response, myeloid cell recruitment and M1 macrophage polarization within the ischemic tissue (90). DC depletion also worsens both mortality and LV deleterious remodeling. DC depletion strongly affects heart healing, with increased myocardial MMP9 (early) and MMP2 (late) activity, on one hand, and more pronounced collagen accumulation in the peri-infarct area, on the other hand. These results suggest that CD11c^+^ DCs play a local protective role in post-infarction inflammation, mainly through a regulation of neutrophil and monocyte recruitment and activation (90). More recently, the distinct roles of plasmacytoid (pDC) and conventional DCs (cDC) in MI have been investigated in mice. pDC depletion using transgenic mice with BDCA2 diphtheria toxin receptor had no impact on cardiac function, whereas depletion of cDC expressing Zbtb46 reduced infarct size and improved systolic cardiac function. cDC depletion was also associated with a reduction of both CD3^+^ T cells and IFN-γ mRNA in the ischemic tissue suggesting that cDC promoted Th1 local responses (88). Conversely, a subset of DC limiting antigen-specific T-cell expansion through nitric oxide synthase 2-dependent nitric oxide production has been reported in a model of autoimmune myocarditis (91). Further studies are required to address the importance of the different DC subsets in heart ischemia and their relevance to human disease.

## Conclusion

A large number of evidence has shown that adaptive immunity is involved in post-ischemic cardiac remodeling in murine models of MI. Future work should aim at characterizing the immune pathways in patients with MI to confirm whether comparable alterations of immune functions contribute to post-ischemic heart dilation and dysfunction. Novel therapeutic strategies aimed at reducing ischemic heart failure development should target the adaptive immune system by either stimulating protective immune functions, including expansion of Tregs, or attenuating the activity of immune pathogenic effectors, including that of Th1, B-2 cells.

## Author Contributions

All authors listed have made a substantial, direct and intellectual contribution to the work, and approved it for publication.

### Conflict of Interest Statement

The authors declare that the research was conducted in the absence of any commercial or financial relationships that could be construed as a potential conflict of interest.
